# Multimodal hand hygiene program: twelve years of continuous improvement in the hospital

**DOI:** 10.1186/2047-2994-4-S1-O21

**Published:** 2015-06-16

**Authors:** JY Kawagoe, AR Toniolo, CM Santos, CV Silva, FG Menezes, HF Castagna, MF Cardoso, P Gonçalves, LG Pontes, L Correa

**Affiliations:** 1 Infection Control Service, Hospital Israelita Albert Einstein, São Paulo, Brazil; 2 Nursing Post-graduation Course , Albert Einstein Nursing Faculty, São Paulo, Brazil

## Introduction

Many organizations and infection prevention experts agree that effective hand hygiene (HH) reduces the incidence of healthcare-associated infections (HAI). Multimodal strategies have been recommended to achieve successful and sustained HH improvement.[1]

## Objectives

Present a twelve-year HH Multimodal Improvement Program at a private and large hospital.

## Methods

Descriptive study about HH compliance improvement and HAI reduction at 650-bed hospital, in São Paulo, Brazil, by implementing multiple actions:

-selecting and installing a good alcohol-based hand rub (ABHR) at point of care with a training program called “Reminder project” (2003-2005);

- providing ongoing training and education and annual campaigns aiming at behavioral change focusing on ABHR as primary product and sustained compliance for HH, using different strategies (formal and web-based training; campaigns with varied themes as “Hospital Safe Attitude”, “Make a Commitment”);

- evaluating and providing feedback on infrastructure and HH compliance, knowledge and perception;

- participating in National and State HH programs (2008 and 2011);

- applying Positive Deviance strategy as a motivational tool for HH promotion;

- achieving leadership and front-line staff commitment and patient and physician engagement.

## Results

ABHR consumption (L/1000 patient-days) increased from 19.0 (2005) to 82.7 (2014) and HH compliance increased from 53.2 % (2008) to 72.6% (2014). HAI incidence density rates per 1000 patient-days decreased from 16.2 (2003) to 4.2 (2014) and central line-associated bloodstream infections density rates reduction from 5.7 (2003) to 1.0 (2014).

## Conclusion

Multimodal HH strategies and leadership engagement were essential to achieve these results. Sustain and improve HH compliance is a continuous challenge.

## Disclosure of interest

None declared.
